# Tissue Regression-Related Alterations in the Expression of Adherens and Tight Junction Proteins in the Hen Oviduct

**DOI:** 10.3390/ijms26199451

**Published:** 2025-09-27

**Authors:** Karolina Frydrych, Anna Hrabia

**Affiliations:** Department of Animal Physiology and Endocrinology, University of Agriculture in Krakow, 31-120 Krakow, Poland; karolina.frydrych@student.urk.edu.pl

**Keywords:** occludin, claudin, JAMs, E-cadherin, β-catenin, tight junction, adherens junction, regression, oviduct, chicken

## Abstract

Intercellular junctions are involved in the regulation of epithelial function and remodeling in the female reproductive system; however, their importance in the avian oviduct is poorly known. The aim of this study was: first, to provide information on the expression and localization of key tight (occludin, claudin 1, 4, 5, junctional adhesion molecule [JAM] 2, 3) and adherens (E-cadherin, β-catenin) junction proteins in the hen oviduct, and second, to compare expression and localization of these molecules between laying and subjected to fasting-induced pause in laying hens. Tissue samples from all oviductal segments, i.e., infundibulum, magnum, isthmus, shell gland, and vagina were collected on the sixth day of the experiment from the control hens and hens that had been fasted for five consecutive days. Specific oviductal part-dependent expression patterns of examined genes (by quantitative real-time polymerase chain reaction [qRT-PCR]) and/or proteins (by Western blotting) were found, with the highest mRNA transcript and protein abundances in the infundibulum, shell gland, and vagina, and the lowest in the magnum. Fasting-induced partial regression of the oviduct was accompanied by alterations in mRNA transcript and protein abundances of examined molecules. Reduced staining intensity of immunoreaction (analyzed by immunofluorescence) for occludin, E-cadherin, and β-catenin proteins was observed in the oviduct of non-laying hens. Our results indicate the potential involvement of these proteins in controlling intercellular communication, cell signaling, paracellular permeability, and mucosal barrier functionality, which impact the functioning of the hen oviduct. Furthermore, our observations provide novel insights into the molecular composition of tight and adherens junctions and its contribution to the remodeling of the oviduct during its regression induced by fasting.

## 1. Introduction

Intercellular communication is fundamental for tissue and organ homeostasis. It facilitates the exchange of signaling molecules, ions, and metabolites, as well as ensures structural cohesion and protects against pathogen intrusion. Three key types of cell–cell junctions are distinguished: tight junctions, adherens junctions, and gap junctions [1,2]. These junctions not only coordinate physiological processes but also govern adhesive interactions critical for maintaining epithelial architecture. The tight and adherens junctions are particularly instrumental in regulating epithelial permeability and reinforcing intercellular adhesion [3,4]. Moreover, these structures play essential roles in tissue remodeling and cell behavior in response to physiological and environmental changes [5,6].

The epithelial lining of the chicken oviduct is a highly specialized structure, characterized by intense secretory activity, responsible for egg formation [7,8]. Its structural integrity may depend on the precise functioning of intercellular junctions, with tight and adherens junction proteins playing pivotal roles.

Tight junctions are complex protein structures that connect epithelial and endothelial cells, forming an intercellular barrier that controls permeability of various substances. They play a crucial role in maintaining epithelial integrity by regulating paracellular transport, i.e., the flow of substances between cells. Tight junctions are involved in many physiological processes, such as nutrient absorption, defense against pathogens, and the regulation of the internal environment [9,10,11]. Their function is dynamically modulated by various factors, including hormonal fluctuations, different cellular signaling pathways, and external stressors. Key molecular components of tight junctions include occludin (*OCLN* gene), claudins (*CLDN*), and junctional adhesion molecules (JAMs) [5,6].

Occludin is one of the key proteins forming tight junctions. It is a transmembrane protein with a molecular weight of approximately 65 kDa, whose function, localization, and interaction with other tight junction proteins such as claudins and JAMs, are tightly regulated by phosphorylation, enabling precise regulation of epithelial barrier properties [12,13]. Occludin has been identified in various tissues, including the uterine epithelium of rats, where it plays an essential role in maintaining barrier integrity and regulating permeability, particularly in preparation for embryo implantation [14]. Furthermore, studies have shown that occludin expression in the rat endometrial epithelium is modulated by testosterone, influencing embryo attachment and implantation [15]. Claudins belong to family of membrane proteins that play a crucial role in regulating paracellular permeability and molecular transitions in the epithelia. These proteins are essential for maintaining epithelial tightness, which influences barrier functions and cell–cell and cell-extracellular matrix adhesion processes [16]. Junctional adhesion molecules, such as JAM 2 and JAM 3, serve as key regulators of cell polarity and intercellular cohesion [17]. These proteins by engaging in intricate interactions with other tight junction components and cytoskeletal elements, reinforce epithelial stability and functional integrity. In the mouse embryo JAMs regulate the timing of blastocyst cavity formation by establishing a paracellular seal of the trophectoderm prior to implantation [18].

Adherens junctions are essential for stabilizing intercellular contacts and actively participate in mechanotransduction, tissue morphogenesis, and intracellular signaling cascades. Among the most important proteins classified as adherens junction components are E-cadherin (*CDH1*) and β-catenin (*CTNNB1*), which play a crucial role in maintaining epithelial integrity and regulating intercellular adhesion processes [19,20].

E-cadherin is a calcium-dependent transmembrane glycoprotein of the classical cadherin family, mediating homophilic cell–cell adhesion [21,22]. It is responsible for epithelial stability and plays a vital role in reproductive tissues by regulating adhesion in the endometrial epithelium, ensuring barrier function, and influencing epithelial–mesenchymal transitions essential for implantation [23]. β-catenin serves as a key intracellular linker, anchoring E-cadherin to the actin cytoskeleton, while concurrently functioning as a pivotal effector of the Wnt signaling pathway, where it modulates transcriptional programs of governing cell proliferation and differentiation.

Various intercellular junction proteins form a network that contributes to tissue remodeling, including reproductive tissues, under both normal and pathological conditions [5,24]. So far, changes in the expression of claudin 1, 3, and 5 have been shown in the hen oviduct during different phases of the reproductive cycle, suggesting a critical role of these proteins for maintaining epithelial barrier function and immune defense [25,26]. The presence of β-catenin in chicken gonads of both sexes during embryonic development and its involvement in the differentiation of Sertoli cells have also been revealed [27]. Moreover, β-catenin mRNA and protein expression was demonstrated in the developing oviduct of chickens and in four oviductal parts of laying hens [28]. In our recent study [29], zona occludens 1 (ZO1, tight junction protein 1) and connexin 43 (Cx43, gap junction protein) expression was altered in the regressing oviduct of the hen during a pause in laying induced by fasting. These findings indicate that ZO1 and Cx43 proteins may play a potential role in controlling cell–cell communication and regulating epithelial integrity and paracellular permeability during oviductal tissue remodeling [29]. Since proteins of cell–cell junctions may have potential implications in the mechanisms underlying oviduct remodeling, egg formation, transportation and oviposition and, consequently, may affect the quality and production of eggs in hens, in this study we examined hypothesis that the expression of other crucial tight junction and adhesion junction proteins changes during the partial regression of the oviduct. Accordingly, the aim of the study was to examine mRNA transcript and protein abundances of major tight junction and adhesion junction molecules, as well as these protein localizations in all oviductal parts of hens subjected to fasting-induced pause in laying. As previously shown, a pause in laying induced by fasting is accompanied by partial regression of the oviductal tissues [29,30,31,32], making this experimental model particularly valuable for understanding epithelial responses to metabolic stress. We suppose that changes in the abundance of cell–cell junction molecules could be important in the context of the effect of environmental physical stressor (fasting) especially on tissues of the reproductive system not only in birds but also in humans and animals. Moreover, the chicken oviductal segments with specific morphology and physiology may constitute the models for searching for universal mechanisms of reproductive tract physiology and diseases.

## 2. Results

As described in our previous study, fasting caused a decrease in the weight of the oviduct of hens by 62% (*p* < 0.001) compared to the control hens. The partial regression of the oviductal tissues of fasted hens was described as well [29].

### 2.1. Messenger RNA Transcript Abundance of Tight Junction Protein Genes (OCLN, CLDN1, CLDN4, CLDN5, JAM2, and JAM3) in the Oviduct of Control and Fasted Hens

**Figure 1:**
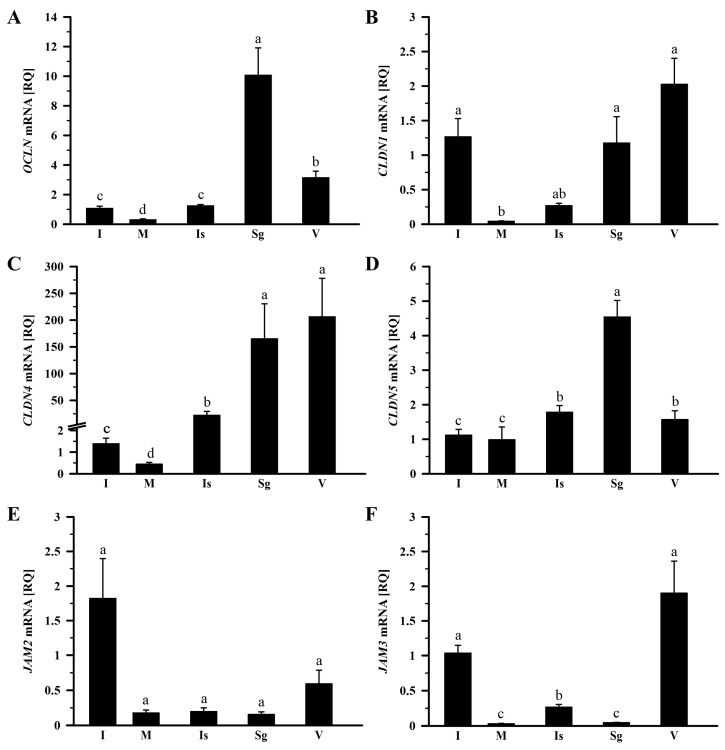
Relative gene expression of *OCLN* (**A**), *CLDN1* (**B**), *CLDN4* (**C**), *CLDN5* (**D**), *JAM2* (**E**), and *JAM3* (**F**) in the oviduct of the control (laying) hens. Each value represents the mean relative quantity (RQ) ± standard error of the mean (SEM) from five or six different hens, normalized to *18S rRNA* expression and standardized to the expression in the infundibulum. Values labeled with different letters differ significantly (*p* < 0.05; Kruskal–Wallis test followed by the Student–Newman–Keuls test). Abbreviations: *OCLN*, occludin; *CLDN*, claudin; *JAM*, junctional adhesion molecule; I, infundibulum; M, magnum; Is, isthmus; Sg, shell gland; V, vagina.

In the control group, the relative expression (RQ) of *OCLN*, *CLDN1*, *CLDN4*, *CLDN5*, and *JAM3* mRNA showed significant variation within the oviduct (*p* < 0.001). The highest level of *OCLN* mRNA transcript was observed in the shell gland, lower by 68.8% in the vagina, by 87.7% in the isthmus, and by 89.4% in the infundibulum, and the lowest in the magnum, where it was lower by 97.1% compared to the shell gland (Figure 1A). The expression pattern of *CLDN1* mRNA exhibited higher levels in the vagina, infundibulum, and shell gland, and lower levels in the isthmus and magnum (*p* < 0.001). Compared to the vagina, *CLDN1* mRNA transcript levels in the magnum and isthmus were lower (*p* < 0.05) by 97.9% and 86.8%, respectively (Figure 1B). In the case of *CLDN4* mRNA transcript abundance, the highest levels were found in the vagina and shell gland, lower in the isthmus and infundibulum, and the lowest in the magnum (*p* < 0.001; Figure 1C). Compared to the vagina, *CLDN4* mRNA transcript abundance was lower (*p* < 0.05) by 89.3% in the isthmus, by 99.3% in the infundibulum, and by 99.8% in the magnum. The expression level of *CLDN5* mRNA was highest in the shell gland, lower by 60.8% in the isthmus, by 65.4% in the vagina, by 75.4% in the infundibulum, and by 78.3% in the magnum (*p* < 0.05; Figure 1D). Transcript abundance of *JAM2* did not differ significantly among oviductal parts (*p* > 0.05; Figure 1E). In the case of *JAM3* mRNA transcript abundance, the highest levels were observed in the vagina and infundibulum, lower in the isthmus, and the lowest in the magnum and shell gland (*p* < 0.001). Compared to the vagina, *JAM3* mRNA transcript abundance was lower (*p* < 0.05) by 86.1%, 97.6%, and 98.5%, respectively, in the isthmus, shell gland, and magnum (Figure 1F).

**Figure 2:**
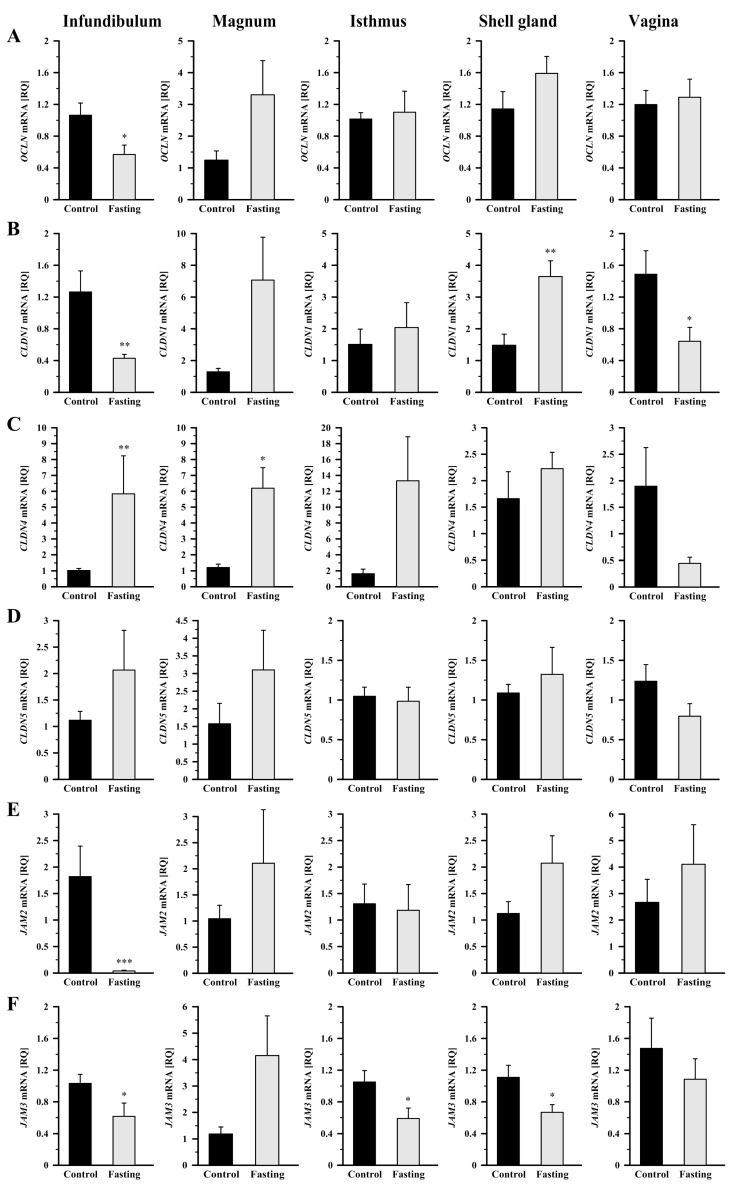
Relative gene expression of *OCLN* (**A**), *CLDN1* (**B**), *CLDN4* (**C**), *CLDN5* (**D**), *JAM2* (**E**), and *JAM3* (**F**) in the oviductal segments of the control and fasted hens evaluated by qRT-PCR. Each value represents the mean relative quantity (RQ) ± SEM from five or six different hens normalized to *18S rRNA* and standardized to the expression in the section of the control hens. * *p* < 0.05, ** *p* < 0.01—compared to the control tissue (Mann–Whitney U test). Abbreviations as in Figure 1.

Compared to control hens, fasting caused a 46.5% decrease (*p* < 0.05) in *OCLN* mRNA transcript abundance in the infundibulum (Figure 2A). In fasted hens, *CLDN1* mRNA transcript abundance was reduced by 66% (*p* < 0.01) in the infundibulum and by 56.8% (*p* < 0.05) in the vagina, whereas it was increased (*p* < 0.01) by 146% in the shell gland, compared to control hens (Figure 2B). Fasting caused an increase in *CLDN4* mRNA transcript abundance in the infundibulum by 473% (*p* < 0.01) and in the magnum by 412% (*p* < 0.05; Figure 2C). Expression of *CLDN5* gene did not change significantly in any oviductal segment (Figure 2D). A pronounced down-regulation in *JAM2* mRNA transcript abundance by 98% was observed in the infundibulum (*p* < 0.001), while no significant differences were found in the other oviductal segments (Figure 2E). *JAM3* mRNA transcript abundance decreased (*p* < 0.05) by 40.4%, 44%, and 40%, respectively, in the infundibulum, isthmus, and shell gland of fasted hens, compared to control hens (Figure 2F).

### 2.2. Messenger RNA Transcript Abundance of Adherens Junction Protein Genes (CDH1, CTNNB1) in the Oviduct of Control and Fasted Hens

**Figure 3:**
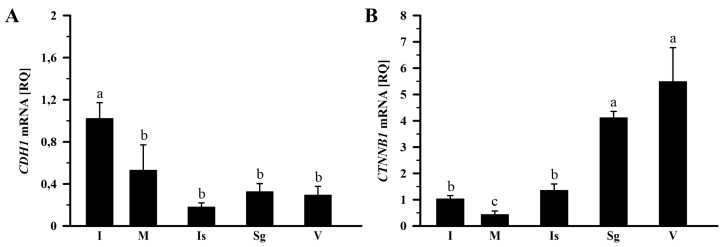
Relative gene expression of *CDH1* (**A**) and *CTNNB1* (**B**) in the oviduct of the control (laying) hens. Each value represents the mean relative quantity (RQ) ± SEM from five or six different hens, normalized to *18S rRNA* expression and standardized to the expression in the infundibulum. Values labeled with different letters differ significantly (*p* < 0.05; Kruskal–Wallis test followed by the Student–Newman–Keuls test). Abbreviations: *CDH1*, cadherin-1; *CTNNB1*, catenin beta-1 (also known as β-catenin). Other abbreviations as in Figure 1.

In the control group, the relative expression (RQ) of *CDH1* mRNA in the infundibulum was higher (*p* < 0.05) than in other parts of the oviduct (Figure 3A), and *CTNNB1* mRNA varied significantly among oviductal segments (Figure 3B). Abundance of *CDH1* mRNA was lower (*p* < 0.05) in the magnum by 48.2%, isthmus by 82.2%, shell gland by 68.3%, and vagina by 71.4% than in the infundibulum (Figure 3A). *CTNNB1* mRNA transcript abundances were the highest in the vagina and shell gland, lower in the isthmus and infundibulum, and the lowest in the magnum (*p* < 0.001). Compared to the vagina, *CTNNB1* mRNA transcript levels in the infundibulum, magnum, and isthmus were lower (*p* < 0.05) by 81.3%, 92.3%, and 75.5%, respectively (Figure 3B).

**Figure 4:**
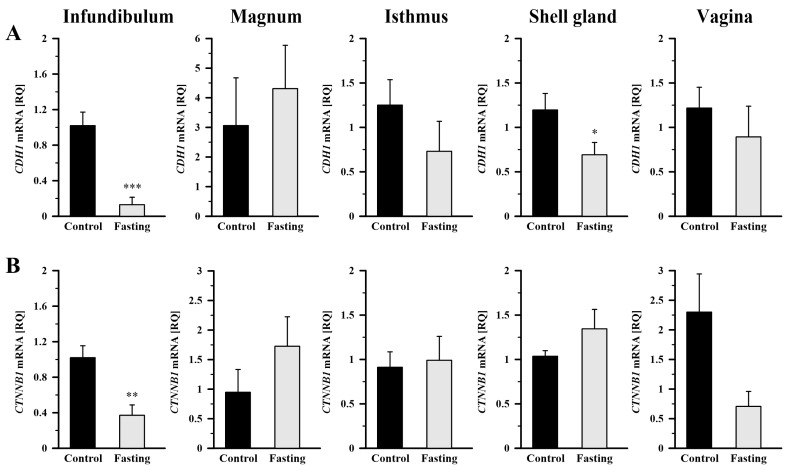
Relative gene expression of *CDH1* (**A**) and *CTNNB1* (**B**) in the oviductal segments of the control and fasted hens evaluated by qRT-PCR. Each value represents the mean relative quantity (RQ) ± SEM from five or six different hens normalized to *18S rRNA* and standardized to the expression in the section of the control hens. * *p* < 0.05, ** *p* < 0.01, *** *p* < 0.001—compared to the control tissue (Mann–Whitney U test). Abbreviations as in Figure 3.

As shown in Figure 4A, fasting led to a significant decrease in *CDH1* mRNA abundance in the infundibulum by 87.3% (*p* < 0.001) and in the shell gland by 57.7% (*p* < 0.05), compared to the control group. In the case of *CTNNB1* mRNA transcript abundance, fasting caused its reduction in the infundibulum by 63.6% (*p* < 0.01) compared to control hens (Figure 4B). Not significant changes were observed in the remaining oviduct segments.

### 2.3. Abundance of Occludin, E-Cadherin, and β-Catenin Proteins in the Oviduct of Control and Fasted Hens

**Figure 5:**
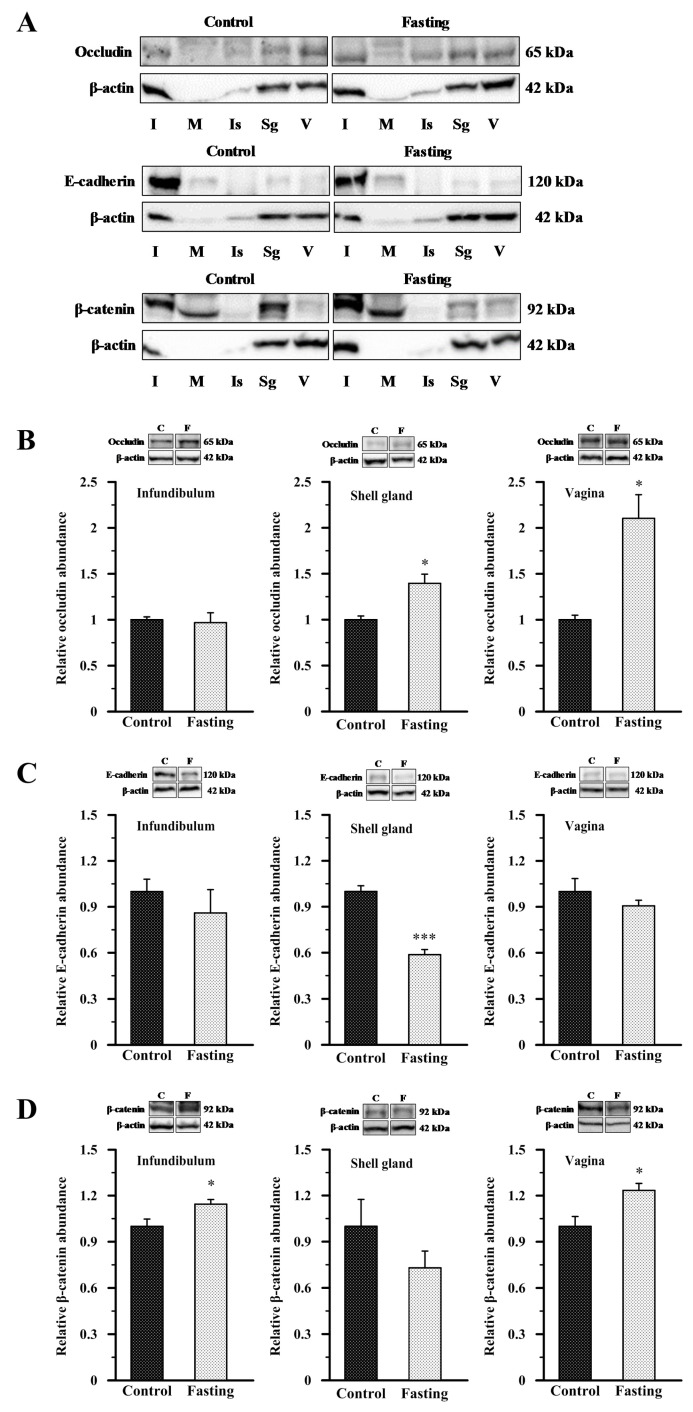
Western blot analysis of occludin, E-cadherin, and β-catenin in the oviduct of control and fasted hens. (**A**) Representative blots of occludin, E-cadherin, and β-catenin in all oviductal segments of the control and fasted birds. (**B**–**D**) The blots and relative abundances of occludin (**B**), E-cadherin (**C**), and β-catenin (**D**) protein in the infundibulum, shell gland, and vagina of the control and fasted hens. Images are representative of at least four independent blots (n = 6–8 different hens/biological replicates) performed for each protein. Graphs depict the relative protein abundances normalized to β-actin. Data are expressed as fold difference ± SEM and compared to the abundance in the control group, considered to be 1. * *p* < 0.05, *** *p* < 0.001—compared to the control tissue (Mann–Whitney U test). Abbreviations as in Figure 1 and Figure 3.

Owing to the limited availability of commercial antibodies for chicken cell–cell junction proteins, we were only able to determine the presence of occludin, E-cadherin, and β-catenin protein in different segments of the chicken oviduct using Western blot (Figure 5). In all oviductual sections, both from control and fasted hens, bands corresponding to molecular weights of approximately 65 kDa, 120 kDa, and 92 kDa were detected for occludin, E-cadherin, and β-catenin, respectively (Figure 5A). Due to the low or undetectable abundance of β-actin in the magnum and isthmus, these segments were excluded from further densitometric analysis. Quantitative analysis of occludin, E-cadherin, and β-catenin protein abundance was conducted in the infundibulum, shell gland, and vagina of both control and fasted hens. In fasted hens, relative abundance of occludin protein was higher by 39.5% (*p* < 0.05) in the shell gland and by 110.5% (*p* < 0.05) in the vagina, compared to control hens (Figure 5B). The abundance of E-cadherin protein was lower by 41.3% (*p* < 0.001) in the shell gland of fasted hens than in control hens (Figure 5C). Fasting also induced a 14.4% increase (*p* < 0.05) in β-catenin protein level in the infundibulum and a 23.4% increase (*p* < 0.05) in the vagina (Figure 5D).

### 2.4. Immunofluorescent Localization of Occludin Protein in the Oviduct of Control and Fasted Hens

**Figure 6:**
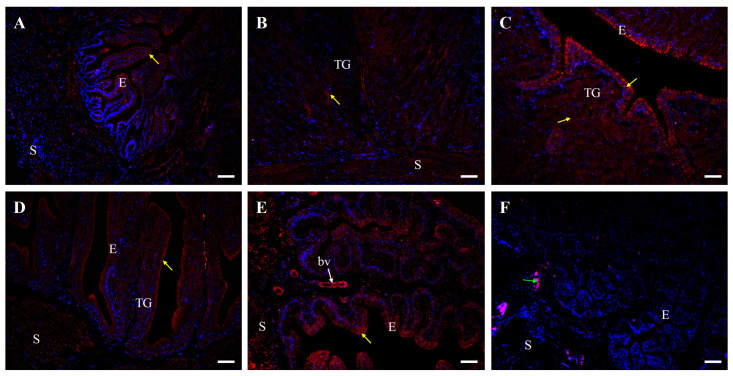
Immunofluorescent localization of occludin protein in all parts of the oviduct: infundibulum (**A**), magnum (**B**), isthmus (**C**), shell gland (**D**), and vagina (**E**) of the control (laying) hens. Positive staining (red fluorescence) is marked with yellow arrows. DAPI staining (blue fluorescence) shows the cell nuclei. Nonspecific staining in erythrocytes is indicated by a green arrow. Moderate occludin immunopositive signals between neighboring cells are observed in the luminal epithelium (E) of the isthmus, shell gland, and vagina, as well as in the stroma (S; muscles + connective tissue) of the vagina and in blood vessels (bv, white arrow). Weak or very weak positive signals, appearing as distinct foci or irregular lines, are detected in the luminal epithelium and muscles of the infundibulum, in the tubular glands (TG) of the magnum, isthmus, and shell gland, and in the muscles of the magnum and shell gland. (**F**) A negative control section incubated without the primary antibody did not exhibit positive reactivity. Scale bars = 50 μm.

**Figure 7:**
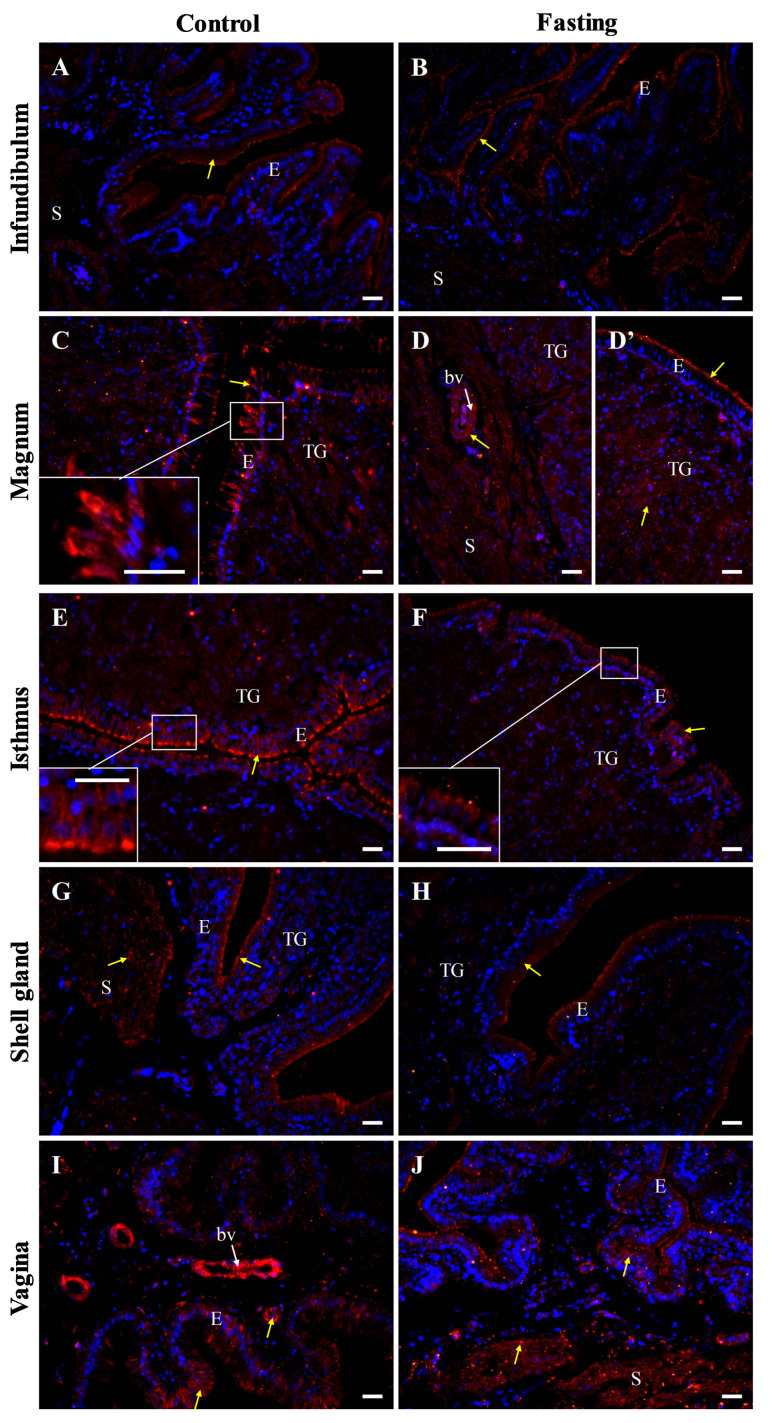
Immunofluorescent localization of occludin protein in oviductal sections of the control (**A**,**C**,**E**,**G**,**I**) and fasted (**B**,**D**,**D’**,**F**,**H**,**J**) hens. Positive signals (red) are indicated by yellow arrows. DAPI staining (blue) shows nuclei. Frames in (**C**,**E**,**F**) indicate the location of a higher magnification view. The blood vessels (bv) are indicated by white arrows. Of note, the intensity of the immunoreactivity for occludin in oviductal parts of non-laying hens is reduced compared to the control hens. Abbreviations: E, luminal epithelium; TG, tubular glands; S, stroma (muscles + connective tissue). Scale bars = 20 μm.

In line with Western blot results, immunofluorescent analysis confirmed the presence of occludin protein in all oviduct segments (Figure 6 and Figure 7). In control hens, moderate positive signals were observed as distinct points or irregular structures between adjacent cells in the luminal epithelium of the isthmus, shell gland, and vagina. Additionally, moderate punctate signals were detected in the vaginal stroma, particularly within smooth muscles interwoven with the connective tissue and in the wall of blood vessels. Weak occludin immunoreactivity was found in the luminal epithelium of the infundibulum, tubular glands of the isthmus and shell gland, as well as in muscles of the magnum and shell gland. In contrast, very weak signals were noted in the tubular glands of the magnum and muscles of the infundibulum (Figure 6).

In fasted hens, reduced intensity of the occludin immunoreactivity was observed, compared to control hens (Figure 7).

### 2.5. Immunofluorescent Localization of E-Cadherin Protein in the Oviduct of the Control and Fasted Hens

**Figure 8:**
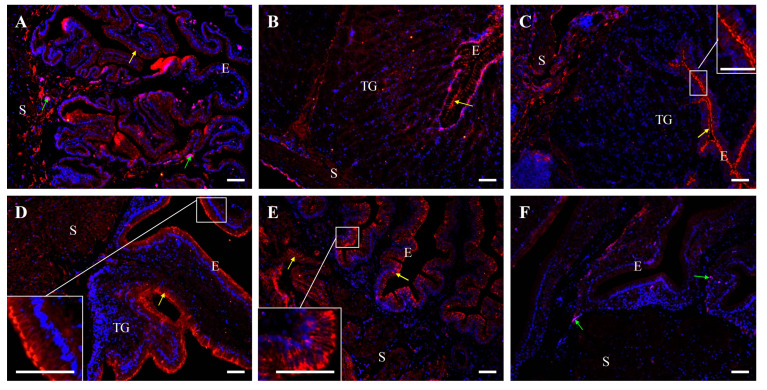
Immunofluorescent localization of E-cadherin protein in all parts of the oviduct: infundibulum (**A**), magnum (**B**), isthmus (**C**), shell gland (**D**), and vagina (**E**) of the control hens. Positive staining (red fluorescence) is marked with yellow arrows. DAPI staining (blue fluorescence) shows the cell nuclei. Non-specific staining in erythrocytes is indicated by green arrows. Frames in (**C**–**E**) indicate the location of a higher magnification view. Strong E-cadherin immunopositive signals are observed in the membranes of neighboring cells in the luminal epithelium (E) of the magnum, isthmus, shell gland, and vagina. Moderate immunopositive reaction is visible in luminal epithelium of the infundibulum, as well as in the stroma (S; muscles + connective tissue) of the infundibulum, shell gland, and vagina. Very weak signals are detected in the tubular glands (TG) of the magnum, isthmus, and shell gland. (**F**) A negative control section incubated without the primary antibody did not exhibit positive reactivity. Scale bars = 50 μm.

**Figure 9:**
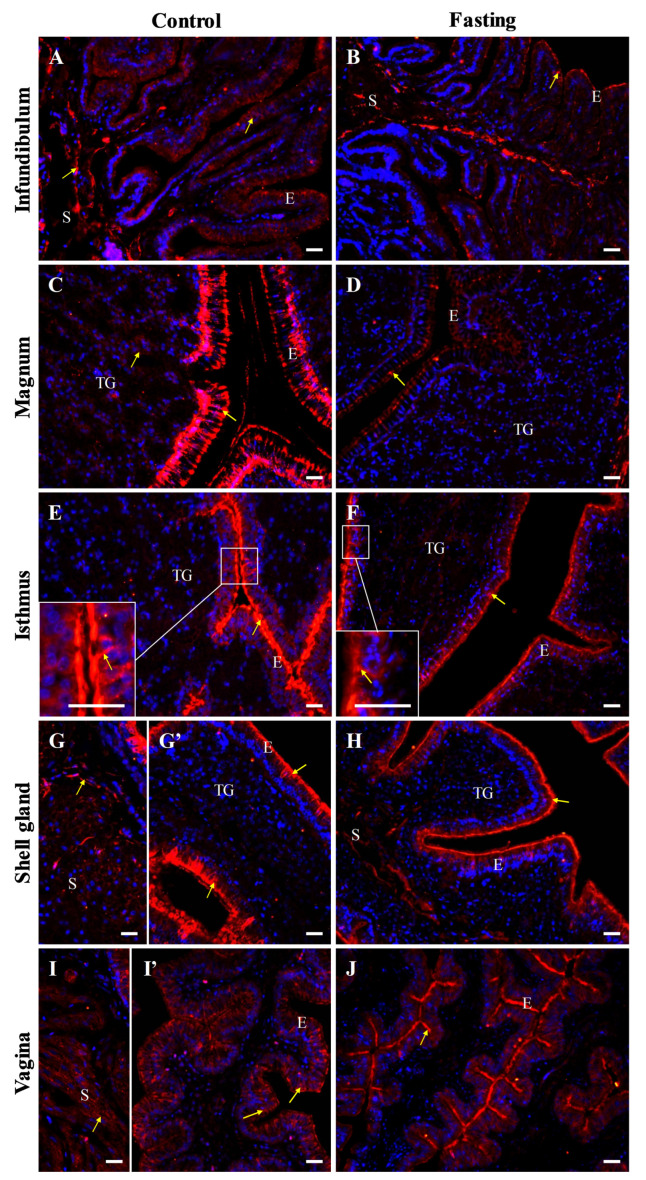
Immunofluorescent localization of E-cadherin protein in oviductal sections of the control (**A**,**C**,**E**,**G**,**G’**,**I**,**I’**) and fasted (**B**,**D**,**F**,**H**,**J**) hens. Positive signals (red) are marked with yellow arrows. DAPI staining (blue) indicates the nuclei. Frames in (**E**,**F**) indicate the location of a higher magnification view. It is noteworthy that the immunoreactivity for E-cadherin in the luminal epithelium of the oviduct is reduced in the non-laying hens compared to the control birds, and the staining is more diffused. Abbreviations as in Figure 7. Scale bars = 20 μm.

Immunofluorescence also confirmed the presence of E-cadherin protein in all segments of the oviduct (Figure 8 and Figure 9). In control hens, strong immunopositive signals for E-cadherin were observed in the membranes of adjacent cells of the luminal epithelium in the magnum, isthmus, shell gland, and vagina. Moderate immunoreactivity was detected in the luminal epithelium of the infundibulum and smooth muscles of the infundibulum, shell gland, and vagina. In contrast, very weak signals were observed in the tubular gland cells of the magnum, isthmus, and shell gland (Figure 8).

In fasted hens, E-cadherin immunoreactivity was distinctly reduced and moved to apical region of epithelial cells across all oviductal segments, compared to control hens (Figure 9). Additionally, the immunostaining pattern appeared more diffuse within the oviductal luminal epithelium of fasted hens (Figure 9).

### 2.6. Immunofluorescent Localization of β-Catenin Distribution in the Oviduct of Control and Fasted Hens

**Figure 10:**
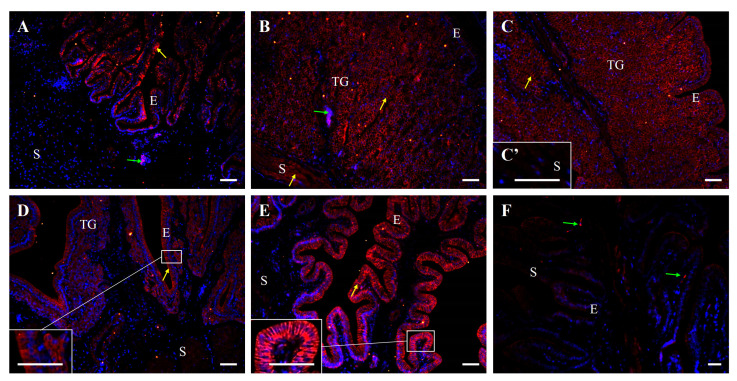
Immunofluorescent localization of β-catenin protein in all parts of the oviduct: infundibulum (**A**), magnum (**B**), isthmus (**C**,**C’**), shell gland (**D**), and vagina (**E**) of control hens. Positive staining (red fluorescence) is marked with yellow arrows. DAPI staining (blue) shows the cell nuclei. Non-specific staining in erythrocytes is indicated by green arrows. Frames in (**D**,**E**) indicate the location of a higher magnification view. Strong β-catenin immunopositive signals are detected in the membrane and cytoplasm of cells in the luminal epithelium (E) of the infundibulum and vagina, as well as in tubular glands (TG) of the magnum and isthmus. Moderate positive signals are detected in the luminal epithelium of the magnum, isthmus, and shell gland, as well as in tubular glands of the shell gland, and in stromal muscles (S; muscles + connective tissue) of the magnum. Weak or very weak immunoreactivity is observed in muscles of the infundibulum, isthmus, shell gland, and vagina. (**F**) A negative control section incubated without the primary antibody did not exhibit specific reactivity. Scale bars = 50 μm.

**Figure 11:**
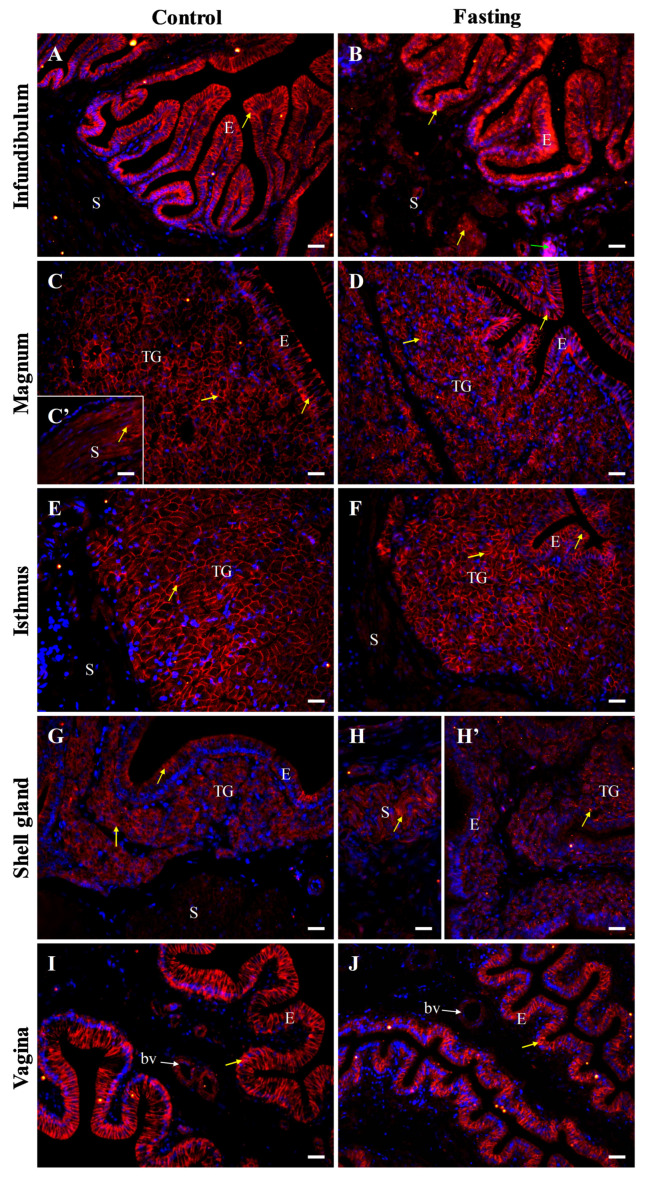
Immunofluorescent localization of β-catenin protein in oviductal sections of the control (**A**,**C**,**C’**,**E**,**G**,**I**) and fasted (**B**,**D**,**F**,**H**,**H’**,**J**) hens. Positive immunoreactivity (red) for β-catenin protein is indicated by yellow arrows. DAPI staining (blue) indicates the nuclei. Non-specific staining in erythrocytes is indicated by green arrow. Blood vessels (bv) are marked with white arrows. Of note, immunoreactivity of β-catenin in the oviduct of the fasted chickens is slightly reduced compared to the control birds. Moreover, in non-laying hens, the signals appear less organized and dispersed. Abbreviations as in Figure 7. Scale bars = 20 μm.

Immunofluorescent analysis further verified the presence of β-catenin protein in all oviductal parts (Figure 10 and Figure 11). In control hens, strong immunopositive signals, visible as distinct clusters or irregular bands, were observed in the cell membrane and in cytoplasm adjacent to the cell membrane in the luminal epithelium of the infundibulum and vagina, as well as in tubular glands of the magnum and isthmus. Moderate β-catenin immunoreactivity was noted in the luminal epithelium of the magnum, isthmus, and shell gland, as well as in the tubular glands of the shell gland, the stromal smooth muscles in the magnum, and blood vessels in the vagina. In contrast, weak or very weak signals were found in stromal muscle cells of the infundibulum, isthmus, shell gland, and vagina (Figure 10).

In fasted hens, β-catenin immunoreactivity was slightly reduced in the shell gland and vagina, compared to the control group. Furthermore, the immunopositive signals appeared more irregular and dispersed, especially in the infundibulum, shell gland, and vagina (Figure 11). In the magnum and isthmus of fasted hens, a reduction in the height of the luminal epithelium and involution of the tubular glands were clearly seen (Figure 11).

Negative control sections did not show immunopositive signals for occludin, E-cadherin, and β-catenin when were incubated without the primary antibody (Figure 6, Figure 8 and Figure 10).

## 3. Discussion

The present study has demonstrated (1) the expression of genes coding crucial tight junction proteins (*OCLN*, *CLDN1*, *CLDN4*, *CLDN5*, *JAM2*, and *JAM3*) and adherens junction proteins (*CDH1* and *CTNNB1*), as well as (2) protein abundance and immunofluorescent localization of occludin, E-cadherin, and β-catenin in oviductal segments of laying and non-laying hens. These results are a substantial extension of our recent findings, i.e., changes in the expression of ZO1 (other tight junction molecule) and Cx43 (gap junction protein) gene and protein in the oviduct of non-laying hens [29]. As documented previously [29], the oviduct of fasted, non-laying hens was characterized by reduced weight and partial tissue regression. Our results presented herein point toward the involvement of key proteins of tight junctions and adherens junctions in the hen oviduct regression, as well as to their potential implication for functioning of the oviduct and the quality of the egg laid by commercially used hens. These results also broaden the suggestion of Ariyadi et al. [25] that tight junction molecules such as claudin 1, 3, and 5 are involved in lower oviductal segments regression during molting in chickens. A previous study by Bae et al. [28] provided evidence of β-catenin expression in the reproductive tract of female chickens, as well as estrogen and several miR regulation of its cell-specific expression during development of the oviduct.

In laying hens, we observed differential abundance of *OCLN*, *CLDN1*, *CLDN4*, *CLDN5*, *JAM2*, *JAM3*, *CDH1*, and *CTNNB1* transcript, as well as occludin, E-cadherin, and β-catenin proteins in all oviductal segments: the infundibulum, magnum, isthmus, shell gland, and vagina. Mostly, the highest abundance of mRNA transcripts for these genes was detected in the infundibulum, shell gland, and/or vagina, and the lowest in the magnum. Similarly, protein abundance of occludin, E-cadherin, and β-catenin was the highest in the infundibulum, shell gland, and vagina. The differences in abundance of mRNA transcripts and proteins between oviductal parts may be related to different tissue composition and function of given segment of the oviduct, as well as different physiological roles of particular proteins of cell–cell junctions. The oviduct of female birds consists of five regions with specific morphology and function in the egg formation. The infundibulum captures the ovum after ovulation and secretes the first layer of the albumen. The magnum secretes the majority of egg white. The isthmus forms the eggshell membranes. The shell gland (uterus) forms the calcified eggshell, and the vagina helps in the expulsion of the egg and is a storage site for spermatozoa in the sperm storage tubules located at the utero-vaginal junction [8]. Moreover, mucosal barrier systems, including epithelial tight junctions, mucin layer, and leukocyte activity in the vagina, have fundamental roles to prevent infection of the oviduct by microorganisms colonizing the cloaca [25].

Our immunofluorescent analysis showed differential intensity of immunoreactive signals of occludin, E-cadherin, and β-catenin protein within the oviductal wall of hens, further indicating significant roles of these proteins in formation of tight and adherens junctions between cells, especially in the luminal epithelium, where they were localized in great abundances. These junctions control paracellular permeability [33] and intercellular adhesion, as well as maintain epithelial integrity and homeostasis [19,20]. In regard to β-catenin, our results are in line with that of Bae et al. [28], who observed strong expression of β-catenin mRNA and protein in the luminal epithelium of four segments (the vagina was not examined) of the chicken oviduct and in the tubular glands (glandular epithelium) of the isthmus and shell gland. We additionally demonstrated strong staining for β-catenin in tubular glands of the magnum and the luminal epithelium of the vagina, where β-catenin as a multitasking protein may play an important physiological role. In addition to acting as a molecular bridge between the cytoplasmic domain of E-cadherin and α-catenin, a portion of cytoplasmic β-catenin is a key player in the Wnt signaling pathway, acting as a transcriptional regulator that controls cell proliferation and differentiation [20,34]. Thus, in the chicken oviduct, β-catenin may also be involved in the regulation of processes related to cell behavior and synthesis of egg components. To our knowledge, the expression of *OCLN*, *CLDN4*, *JAM2*, *JAM3*, and *CDH1* genes and occludin and E-cadherin proteins in all segments of the avian oviduct was shown for the first time in this study. These novel findings indicate the participation of mentioned molecules in tight junction or adherens junction assembly and maintenance of tissue homeostasis in the hen oviduct. The presence of several tight junction and adherens junction proteins was previously demonstrated in the oviduct and/or uterus of mammalian species, e.g., rats, mice, dogs, cattle, and pigs [14,26,35,36,37,38,39,40], which are engaged in fertilization and embryo–maternal interactions. Likewise, cell–cell junction proteins may play significant role in specific functions of a given segment of the avian oviduct, including the maintenance of tissue integrity and mechanical barrier against pathogens, as well as regulating the paracellular transport of substances and influencing signaling pathways, which determine the cell functioning related to the egg formation. Of particular interest is the great expression/abundance of examined tight and adherens junction proteins in the infundibulum, shell gland, and vagina, which were localized principally in the luminal epithelium. Since tight junctions control the movement of solutes along the paracellular pathway [6], we propose that tight junction-associated proteins might participate in the controlling of fluid secretion, creating a specific microenvironment for the ovum fertilization in the infundibulum, eggshell biomineralization in the shell gland, and sperm storage in sperm storage tubules in the utero-vaginal junction. Interestingly, moderate or weak immunoreactivity for occludin, E-cadherin, and β-catenin proteins were also localized to the smooth muscles within the oviductal stroma. This is in line with the distribution of ZO1 protein in the hen oviduct [29], pointing out that key tight junction and adherens junction proteins may takes part in maintaining oviductal muscle integrity and coordination of myometrial contractions essential for egg moving along the oviduct.

Numerous studies have highlighted the impact of cell–cell junctions to tissue remodeling under physiological and pathological conditions [20,41,42]. Defects in cell–cell junctions give rise to a wide range of abnormalities that disrupt tissue homeostasis [20]. In hens, disorders in the function of the reproductive system directly affect the quality and number of eggs laid. A previous report indicates that the mucosal barrier in the lower oviductal segments is disrupted in non-laying hens during molting due to reduced claudin expression [25]. Moreover, there are changes in Cx43 and ZO1 gene and/or protein expression in a partly regressed chicken oviduct after fasting or treatment with an estrogen receptor blocker [29,43]. Therefore, further in this study, we focus on the examination of the expression and localization of several proteins involved in tight and adherens junction establishment in the regressing oviduct of hens. We found that partial regression (described deeply in a previous study of Frydrych et al. [29]) of the oviduct was accompanied by alterations in the mRNA transcript abundance of tight and adherens junction molecules, and their expression levels depended on the oviductal segment and type of gene. The changes were observed primarily in the infundibulum and shell gland. In most cases, the mRNA transcript abundance was reduced in the oviduct of fasted hens, compared to control hens, but upregulation of tight junction protein genes was also observed. That differential expression of genes in oviductal parts of hens might be attributable to the diverse response of given gene and oviductal part (with different cell composition) to fasting. Further, we observed reduced protein abundance of E-cadherin in the shell gland, which coincides with lowered abundance of this protein mRNA transcript in fasted hens. On the contrary, protein abundance of occludin in the shell gland and vagina and β-catenin in the infundibulum and vagina was elevated in non-laying hens, compared to laying hens. These discrepancies between the mRNA and protein abundances in some tissues might be related, at least in part, to posttranscriptional regulatory mechanisms. Increased abundance of mRNA transcript and protein in partly regressed oviduct of hens was also demonstrated for ZO1 [29]. Opposite observations were made by Ariyadi et al. [25], who demonstrated a prominent downregulation of claudin 1, 3, and 5 genes in the oviduct of molting hens. The differences between our results and those previously demonstrated might be attributable to the type of protein examined and/or the degree of oviductal tissue regression. It should be noted that in the study of Ariyadi et al. [25], the regression of the oviduct was much more advanced than in our study. Nevertheless, observed alterations in mRNA transcript and/or protein abundance of crucial tight junction (occludin, claudin 1, 4, JAM2, JAM3) and adherens junction (E-cadherin, β-catenin) proteins clearly indicate that these proteins may impact on the formation of tight and adherens junctions, as well as affect other cell junctions (e.g., gap junctions) and cell signaling pathways (e.g., Wnt signaling). Subsequently, these changes can impair functionality of oviductal epithelium (luminal and glandular) in chickens. We hypothesize that alterations in the abundance and distribution of intercellular junction proteins might serve as markers of mucosal damage in the avian reproductive tract and other animals in response to environmental stress, food deprivation, parasite invasion, and/or numerous untreated diseases.

It should be stressed that alterations in tight and adherens junction functionality can occur not only via the modulation of gene expression of proteins forming these junctions but also through protein phosphorylation, cleavage, cellular relocation, or complex interactions with other proteins [20,44,45,46]. For example, tight junction organization and function are regulated by phosphorylation status of occludin, claudins, and ZO1. Mislocalization of ZO1 may be caused by the lack of claudin recruitment and formation of tight junctions, which ultimately results in epithelial barrier disruption [47]. Balance between sequestration and the release of β-catenin from E-cadherin is important in regulating cell proliferation and differentiation [20]. Redistribution of E-cadherin is required during cell migration [48]. Moreover, shedding of the E-cadherin extracellular domain mediated by the matrix metalloproteinase (MMP) eliminates its residual adhesive activity and results in a complete disruption of cadherin-mediated cell–cell adhesion [44]. For an effective interplay among cell–cell adhesion, detachment, proliferation, and survival under different physiological and pathological conditions, the coordinated processing of E-cadherin by a disintegrin and metalloproteinase 10 (ADAM10) may also be significant [45]. To establish the precise roles of mentioned modifications of cell–cell junctional proteins and their importance in the avian oviduct regression/remodeling, further experiments are necessary. In particular, the use of techniques for functional knockout of genes and their products appears to be valuable for elucidating the role of each specific gene in the oviductal parts.

Of particular interest in this study was the observation of reduced immunoreaction intensity and alterations in the signal patterns for occludin, E-cadherin, and β-catenin proteins in the regressing oviduct of non-laying hens compared to laying hens. Specifically, for occludin, decreased immunoreactivity in the shell gland and vagina of fasted hens contrasted with its abundance estimated by Western blot. The discrepancy between the abundance of occludin observed in both analyses may be attributed, at least in part, to tissue processing, antibody applicability for a given technique, and the specificity and sensitivity of the techniques used. As described previously [29] and observed herein, fasting caused partial regression of the oviduct with degeneration of the luminal epithelium, as well as disorganization and involution of the tubular glands. This regression state of the oviduct was characterized by irregular and/or more diffused immunopositive signals for occludin, E-cadherin, and β-catenin. Moreover, immunoreactivity for E-cadherin protein was moved to apical side of epithelial cells along the oviduct. Thus, it is tempting to speculate that changes in oviductal tissue structure and activity [29], including impaired epithelial barrier functionality [25], are linked to disruption in molecular composition of cell–cell junctions, in which occludin, E-cadherin, and β-catenin contribute. This may alter mucosal tissues’ susceptibility to pathogen invasion, intercellular communication, paracellular diffusion, and/or cell fate, including apoptosis. These observations also emphasize the great importance not only of the appropriate level of expression of cell–cell junction proteins but also their location. During fasting-induced pause in laying, numerous apoptotic cells appear in the oviduct [49], and morphological changes observed during apoptosis, at least in part, result from effects on cell–cell connections. The cadherin–catenin adhesion complex, one of the major adhesive system in multiple epithelial tissues, is targeted during apoptosis [50,51] by caspase 3 and MMPs. These enzymes efficiently cleave the β-catenin and E-cadherin in epithelial cells [44]. It is interesting to note here that increased expression and activity of MMP 2, 7, and 9 are observed in the chicken oviduct during fasting-induced pause in laying [30].

The oviduct of birds is a steroid hormone-responsible organ, and during natural or fasting-induced pause in laying, sex steroid concentrations in the blood plasma and partly regressed oviductal tissues markedly decrease [31,52,53]. Thus, the hen oviduct can constitute a unique model to study the oviduct and uterus physiology and diseases based on hormonal background, including uterine tumors. It is becoming increasingly evident that occludin, claudins, JAMs, β-catenin, and/or E-cadherin are regulated by gonadal steroids in avian and/or mammalian species [9,10,11,14,15,25,28,36,39]. Therefore, altered abundance of *OCLN*, *CLDN1*, *CLDN4*, *JAM2*, *JAM3*, *CDH1*, and *CTNNB1* transcripts and occludin, E-cadherin, and β-catenin proteins, as well as reduction in occludin, β-catenin, and E-cadherin immunoreactivity in some parts of the oviduct of the fasted hens compared with the control hens may be attributable to limited action of ovarian steroids. The influence of estrogen on claudin [25] and β-catenin [28] expression in the chicken oviduct was demonstrated, but the impact of ovarian steroids in orchestration of the expression of other mentioned constituents of tight and adherens junctions remains to be studied in vivo and in vitro.

## 4. Materials and Methods

### 4.1. Animals and Tissue Collection

Hy-Line Brown laying hens were obtained from a commercial supplier and housed individually under controlled conditions, with a 14 h light and 10 h dark photoperiod. Throughout the experiment, the hens had unrestricted access to commercial feed and water. Oviposition time was recorded daily for each hen, allowing the determination of individual laying cycles. Based on a previously established protocol [29,30,31,32,54], at 32 weeks of age, hens were randomly assigned to two experimental groups: (1) a control group fed ad libitum (C; n = 8) and (2) a fasted group subjected to an induced pause in laying through complete feed withdrawal for 5 days (F; n = 8). The hens of the fasted group terminated their egg laying, on average, on day 3.7 ± 0.31 of the experiment. All hens were euthanized on day 6 of the experiment. Control hens that laid eggs were sacrificed 2 h after oviposition, when the egg was located in the magnum of the oviduct. The study was conducted following ethical guidelines, with regular monitoring of animal health and welfare to minimize stress and discomfort. The oviduct was carefully dissected. Then, tissue samples from the central portion of each oviductal segment (the infundibulum, magnum, isthmus, shell gland, and vagina) were either flash-frozen in liquid nitrogen and stored at −80 °C for subsequent Western blot analysis or preserved in RNAlater (Sigma-Aldrich, Saint Louis, MO, USA) and kept at −20°C for later total RNA isolation and quantitative real-time PCR analysis (qRT-PCR). Additional tissue samples were fixed in 10% buffered formalin, dehydrated through a graded ethanol series, cleared in xylene, and embedded in paraffin wax. Sections of 6 μm thickness were obtained using a microtome, mounted on glass slides, and used for immunohistochemical analysis.

### 4.2. Total RNA Isolation, Reverse Transcription, and Quantitative Real-Time PCR

RNA isolation and reverse transcription (RT) were performed according to the protocols established by Hrabia et al. [43], while qRT-PCR analyses were conducted according to Proszkowiec-Weglarz et al. [55], Grzegorzewska et al. [56], and Frydrych et al. [29]. In brief, total RNA was extracted from tissue samples using the TRI reagent (Sigma-Aldrich). Then, 2 μg of total RNA from each sample were reverse-transcribed using a high-capacity cDNA reverse transcription kit containing random primers (# 4368814; Thermo Fisher Scientific, Waltham, MA, USA). The samples were incubated in a thermocycler (Mastercycler Gradient, Eppendorf, Hamburg, Germany) following the manufacturer’s recommended protocol: 10 min at 25 °C, 120 min at 37 °C, and 5 min at 85 °C. The resulting complementary DNA (cDNA) was used in qRT-PCR for multiple target genes, with *18S ribosomal RNA* (*18S rRNA*; reference gene) serving as the normalization control, in a 96-well thermocycler (StepOne Plus; Applied Biosystems, Foster City, CA, USA). The cycling program was set as follows: 15 min at 95 °C, followed by 40 cycles of 15 s at 95 °C, 20 s at 64 °C, and 20 s at 72 °C.

Primer sequences for the target genes were designed based on previously validated sequences (Table 1). Singleplex qRT-PCR reactions were prepared in a total volume of 10 μL, consisting of 2 μL of 5 × HOT FIREPol EvaGreen qPCR Mix Plus (Solis BioDyne, Tartu, Estonia), 0.25 μL (or 0.125 μL for *18S rRNA*) of each primer (forward and reverse; at a concentration of 10 pM/μL), 1 μL of cDNA (10-fold dilution post-RT), and PCR-grade water to reach a final volume of 10 μL. Each sample was analyzed in duplicate, and a no-template control was included in each run. The relative quantification of the target genes was determined after normalization with the *18S rRNA* transcript and using expression level from the infundibulum (for comparison among oviduct segments, Figure 1 and Figure 2) or the oviductal segment of control hens (when compared between control and experimental hens; Figure 3 and Figure 4) as a calibrator, employing the 2−ΔΔCt method [57].

### 4.3. Protein Isolation and Western Blot Analysis

To evaluate the abundance of occludin, E-cadherin, and β-catenin proteins, oviductal tissues were homogenized in an ice-cold lysis buffer. Then, samples were vigorously vortexed multiple times and then subjected to sonication. The resulting lysates were centrifuged at 10,000× *g* for 20 min at 4 °C. Protein concentrations in the supernatants were quantified using the Bradford assay, with the Pierce Detergent Compatible Bradford Assay Reagent (Thermo Scientific, Rockford, IL, USA), with bovine serum albumin serving as a standard. According to a previously established protocol [29,43,60], tissue samples containing 60 μg of total protein were combined with loading buffer (Bio-Rad, Hercules, CA, USA), denatured at 99 °C for 7 min, and then loaded onto a sodium dodecyl sulfate-polyacrylamide gel dedicated to the individual protein: occludin was resolved on an 12% gel, E-cadherin on a 10% gel, and β-catenin on an 8% gel. After electrophoretic separation of the proteins under reducing conditions using a Mini-Protean Tetra Cell apparatus (Bio-Rad), the proteins were transferred to a PVDF membrane using a semi-dry blotter with FLASHBlot transfer buffer (Advansta, San Jose, CA, USA) at a constant voltage of 25 for 7 min. The membranes were then blocked for 1 h in Tris-buffered saline (TBS) containing 0.1% (*v*:*v*) Tween 20 (TBST, pH 7.6) and 5% nonfat milk. After washing, the membranes were incubated overnight at 4 °C with primary polyclonal rabbit antibodies specific for occludin, E-cadherin, or β-catenin (specific dilutions and sources detailed in Table 2). The specificity of the primary antibodies was previously confirmed in avian tissues [61]. All antibodies were commercially available and recommended for use in chickens. After incubation with primary antibodies, membranes were washed with TBST and then incubated with goat anti-rabbit secondary antibody conjugated to horseradish peroxidase (HRP) for 1 h. Proteins of interest were detected using enhanced chemiluminescence (WesternBright™ ECL; Advansta), and images were captured with a ChemiDoc-It 410 imaging system using Vision-Works Life Science software, version 6.6a (Ultra-Violet Products Ltd., Cambridge, UK). All immunoblots were then incubated with stripping buffer for 30 min and re-incubated with anti-β-actin antibody for 1 h, which served as a loading control. The experiments were performed in quadruplicate. The bands corresponding to occludin, E-cadherin, and β-catenin, were densitometrically analyzed using ImageJ, version 1.8.0 (developed by the National Institutes of Health, Bethesda, MD, USA) and normalized to the corresponding β-actin bands.

### 4.4. Immunofluorescence

An immunofluorescence assay for the localization of occludin, E-cadherin, and β-catenin protein in individual oviductal parts was carried out following a standardized protocol [29,43,60]. Oviductal tissue sections were deparaffinized in xylene, rehydrated through a graded series of alcohols, washed in water, and subjected to antigen retrieval by microwaved in citrate buffer (pH 6.0, 3 min). To block non-specific binding of the secondary antibody, the sections were incubated with 5% (*v*/*v*) normal goat serum in TBST at room temperature for 30 min. Following blocking, the sections were incubated overnight at 4 °C with polyclonal rabbit antibodies against occludin, E-cadherin, and β-catenin (Table 2). After washing with TBS, the sections were incubated for 90 min with DyLight 594-conjugated anti-rabbit IgG. Subsequently, sections were washed again in TBS and mounted with VECTASHIELD^®^ Vibrance™ Antifade Mounting Medium containing DAPI (Vector Laboratories). Non-specific staining was controlled by replacing the primary antibody with normal rabbit serum or TBST. The tissue sections were analyzed using an Axio Scope.A1 fluorescence microscope, and images were captured using an Axiocam 503 color camera and ZEN 2.3 pro software (Carl Zeiss, Jena, Germany). Immunoreactivity intensity was categorized as strong, moderate, weak, and very weak. Micrographs display merge of blue fluorescence indicating DAPI-stained cell nuclei and red fluorescence indicating immunopositive reaction specific for occludin, E-cadherin or β-catenin.

### 4.5. Statistical Analysis

The Shapiro–Wilk test was used to verify whether the data followed a normal distribution. Homogeneity of variance was assessed with the Brown–Forsythe test. Data of relative gene expression in the oviduct of the control group (Figure 1 and Figure 2) was analyzed using the nonparametric Kruskal–Wallis test, followed by the Student–Newman–Keuls test. The nonparametric Mann–Whitney U test was applied to compare the means of the two groups (fasted vs. control; Figure 3, Figure 4 and Figure 5). Differences in values were considered significant at the 95% confidence level (*p* < 0.05). Calculations were performed using SigmaPlot_V13 (Systat Software Inc., San Jose, CA, USA). Results are presented as the mean ± standard error of the mean (SEM).

## 5. Conclusions

Taken together, the alterations in tight (occludin, claudins, JAMs) and adherens (E-cadherin, β-catenin) junction protein gene expression, immunostaining intensity, and tissue distribution were demonstrated in oviductal segments of non-laying hens compared to laying hens. These results, showed in large part for the first time, point out the potential involvement of these proteins in controlling cell–cell communication, cell signaling, paracellular permeability, and mucosal barrier functionality, which impact the functioning of the hen oviduct during its partial regression induced by fasting. Furthermore, our results provide novel insights into the molecular composition of intercellular junctions in the hen oviduct, and its contribution to the remodeling of the oviductal tissues, as well as emphasize the importance of metabolic state in maintaining the epithelial integrity and functionality in the hen oviduct. Understanding of cell–cell junction protein engagement in mechanisms underlying oviduct functioning and formation of normal egg in female birds may be of considerable significance for poultry production.

## Figures and Tables

**Table 1:** GeneBank accesion numbers, sequences of amplified gene primers used in qRT-PCR assay in hens, amplicon lengths, and references.

Gene	GenBank Accession No.	Primers	Amplicon Size (bp)	References
*CDH1*	NM_001039258	F: 5′-TGGAGCCGGGCGAGTACAATATCTT-3′ R: 5′-TAGTCGAACACCAGCAGCGAGTCGTA-3′	532	[58]
*CLDN1*	AY750897	F: 5′-GACTCGCTGCTTAAGCTGGA-3′ R: 5′-AAATCTGGTGTTAACGGGTG-3′	276	[25]
*CLDN4*	AY435420	F: 5′-ATCGCCCTGTCCGTCATC-3′ R: 5′-ACCACGCAGTTCATCCACAG-3′	137	[55]
*CLDN5*	NM_204201	F: 5′-AGGTGTCAGCCTTCATCGAC-3′ R: 5′-CCAGGATGGAATCGTACACC-3′	123	[55]
*CTNNB1*	NM_205081.3	F: 5′-TATCCCACGGCTAGTTCAGC-3′ R: 5′-TACAGCCCTCAACGATTTCC-3′	125	[27]
*JAM2*	XM_015299112	F: 5′-CTGCTCCTCGGGTACTTGG-3′ R: 5′-CCCTTTTGAAAATTTGTGCTTGC-3′	135	[55]
*JAM3*	XM_417876	F: 5′-CCAGAGTGTTGAGCTGTCCT-3′ R: 5′-AGAATTTCTGCCCGAGTTGC-3′	147	[55]
*OCLN*	NM_205128	F: 5′-GATGGACAGCATCAACGACC-3′ R: 5′-CTTGCTTTGGTAGTCTGGGC-3′	142	[55]
*18S rRNA*	L21170.1	F: 5′-GAGCTCTTTCTCGATTCCGTGGG-3′ R: 5′-GCCAGAGTCTCGTTCGTTATCGG-3′	96	[59]

Abbreviations: *CDH1*, E-cadherin; *CLDN*, claudin; *CTNNB*, beta-catenin; *JAM*, junctional adhesion molecule; *OCLN*, occludin; *rRNA*, ribosomal RNA.

**Table 2:** Primary and secondary antibodies used in Western blot (WB) and immunofluorescence (IF).

Antibody	Serum	Host Species	Vendor	Cat. no	WB Dilution	IF Dilution
Beta-catenin	NGS	Rabbit	Invitrogen, Carsband, CA, USA	71-2700	1:1000	1:75
E-cadherin	NGS	Rabbit	Invitrogen, Carsband, CA, USA	PA5-19479	1:1000	1:100
Occludin	NGS	Rabbit	Invitrogen, Carsband, CA, USA	71-1500	1:250	1:75
Goat anti-rabbit HRP	-	Goat	Advansta Inc., San Jose, CA, USA	R-05072-500	1:5000	-
Anti-beta-actin HRP	-	Mouse	Sigma-Aldrich, St. Louis, MO, USA	A2228	1:500	-
DyLight 594-anti rabbit	-	Goat	Vector Laboratories, Burlingame, CA, USA	DI-1594-1.5	-	1:150

Abbreviations: HRP, horseradish peroxidase; NGS, normal goat serum.

## Data Availability

The raw data used for the preparation of the presented results are available on request from the corresponding author.
